# Increased ShTAL1 IgE responses post-Praziquantel treatment may be associated with a reduced risk to re-infection in a Ghanaian *S*. *haematobium*-endemic community

**DOI:** 10.1371/journal.pntd.0010115

**Published:** 2022-03-09

**Authors:** Elias K. Asuming-Brempong, Irene Ayi, William van der Puije, Ben A. Gyan, Irene A. Larbi, Yvonne Ashong, Naa Adjeley Frempong, Joseph K. Quartey, Joseph Otchere, Frances M. Jones, Shona Wilson, David W. Dunne, Daniel A. Boakye

**Affiliations:** 1 Department of Parasitology, Noguchi Memorial Institute for Medical Research, College of Health Sciences, University of Ghana, Accra, Ghana; 2 Department of Immunology Noguchi Memorial Institute for Medical Research, College of Health Sciences, University of Ghana, Accra, Ghana; 3 Department of Epidemiology, Noguchi Memorial Institute for Medical Research, College of Health Sciences, University of Ghana, Accra, Ghana; 4 Department of Pathology, University of Cambridge, Cambridge, United Kingdom; George Washington University School of Medicine and Health Sciences, UNITED STATES

## Abstract

**Background:**

Evidence from recent studies in *Schistosoma mansoni*-endemic areas show an age-associated immunity that is positively correlated with IgE titres to *Schistosoma mansoni*-specific tegumental allergen-like protein 1 (SmTAL1). The structural homology between SmTAL1 and the *S*. *haematobium*-specific TAL1 (ShTAL1) has been verified, yet it remains unclear whether similar age- and immune-associated trends characterize ShTAL1. This community-based intervention study was conducted to assess whether ShTAL1IgE responses post-treatment with praziquantel (PZQ) might be associated with a reduced risk to re-infection with *S*. *haematobium*.

**Methodology/Principal findings:**

This study was conducted at Agona Abodom, Central Region, Ghana, and involved 114 participants aged 6 to 55 years. EDTA blood samples were collected at baseline and 7 weeks after PZQ treatment (Follow-up). Baseline and Follow-up titres of specific IgG1, IgG4, and IgE antibodies to the *S*. *haematobium*-specific adult worm antigen (ShAWA), the Sh-specific soluble egg antigen (ShSEA), and the Sh-specific tegumental-allergen-like 1 protein (ShTAL1) in plasma samples were measured using sandwich ELISA. Participants at both time points also provided stool and urine for helminth egg detection by microscopy. Prevalence of *S*. *haematobium* at baseline was 22.80%, and decreased to 3.50% at Follow-up. The egg reduction rate (ERR) was 99.87%. Overall plasma levels of ShTAL1-IgE increased 7 weeks post-PZQ treatment, and with increasing age; whiles *S*. *haematobium* infection prevalence and intensity decreased. For *S*. *haematobium*-infected participants who were egg-negative at Follow-up (N = 23), minimal median levels of ShTAL1-IgE were observed for all age groups prior to treatment, whilst median levels increased considerably among participants aged 12 years and older at Follow-up; and remained minimal among participants aged 11 years or less. In the univariate analysis, being aged 12 years or older implied an increased likelihood for ShTAL1-IgE positivity [12–14 years (cOR = 9.64, 95% CI = 2.09–44.51; p = 0.004); 15+ years (cOR = 14.26, 95% CI = 3.10–65.51; p = 0.001)], and this remained significant after adjusting for confounders [12–14 years (aOR = 22.34, 95% CI = 2.77–180.14; p = 0.004); ≥15 years (aOR = 51.82, 95% CI = 6.44–417.17; p < 0.001)]. Conversely, median ShTAL1-IgG4 titres were hardly detectible at Follow-up.

**Conclusions/Significance:**

These findings demonstrate that increased IgE levels to ShTAL1 7 weeks after PZQ treatment could be associated with a reduced risk to re-infection, and adds to the large body of evidence suggesting a protective role of the treatment-induced ShTAL1 antigen in schistosomiasis infections. It was also quite clear from this work that apart from being persistently *S*. *haematobium*-positive, elevated ShTAL1-IgG4 levels at Follow-up could be indicative of susceptibility to re-infection. These outcomes have important implications in vaccine development, and in shifting the paradigm in mass chemotherapy programmes from a ‘one-size-fits-all’ approach to more sub-group-/participant-specific strategies in endemic areas.

## Author summary

The current World Health Organization (WHO)—approved strategy for combating schistosomiasis, is the administration of praziquantel (PZQ) to subjects living in endemic communities. Due to concerns of the potential development of resistance to PZQ, there have been extensive studies to find putative candidate antigens capable of eliciting protective immunity particularly against the schistosomulum. One of such family of antigens discovered is the *Schistosoma mansoni* tegumental allergen-like proteins 1 through 13 (SmTAL1–13), of which the first six have been extensively studied. Although not present in the 3-hour schistosomulum, the SmTAL1 antigen has been found to induce cross-reactive IgEs that also recognize the SmTAL3 and SmTAL5 antigens present on the surface of cercariae and schistosomulae. Various epidemiological studies have indicated IgEs induced by SmTAL1 as potentially good markers/indicators for developing resistance to infection/re-infection. In this community-based intervention study, we sought to determine whether outcomes realized for *Schistosoma haematobium* TAL1 IgE. 7 weeks-post PZQ treatment would be predictive of protection against re-infection with *S. haematobium*. Our work not only assesses immune responses associated with ShTAL1, but also explores immune profiles that may characterize participants susceptible or resistant to re-infection with *S. haematobium*.

## Introduction

Schistosomiasis, a major public health concern in many low-to-middle-income countries (LMICs), comes second to malaria in its human health impact [1]. Globally, 207 million people are infected, while 20 million suffer from the chronic form of the disease [2]. In sub-Saharan Africa, urogenital schistosomiasis caused by *S*. *haematobium*; and intestinal schistosomiasis caused by *S*. *mansoni* and *S*. *intercalatum*, are highly endemic. Indeed, *S*. *haematobium* and *S*. *mansoni* are most widely distributed in many communities in Ghana, causing a significant burden of mortality [3–5].

In schistosomiasis-endemic areas, infection may be life-long. However, the likelihood for re-infection reduces with ageing, suggesting the gradual development of acquired resistance to re-infection [6,7]. The likelihood and intensity of re-infection after chemotherapy is higher amongst children and adolescents than among adults, who develop immunity targeted presumably at vulnerable stages of the parasite’s life cycle, such as, the skin stage schistosomula. Epidemiological studies have correlated human IgE responses against the schistosomula or adult-worm with immunity. This is due to the gradual increase in observed IgE titres to the worm antigens which correlates with the age-dependent immunity seen in endemic areas [7,8]. In monitoring re-infection post-chemotherapy, it has been shown that people with high levels of parasite-specific IgE are less likely to become re-infected [9,10]. This, according to a number of *S*. *mansoni*-related field studies, is due to the death of the adult worm following chemotherapy, which introduces large amounts of schistosome antigens in circulation, leading to the priming and boosting of IgE levels [11,12].

A number of immuno-epidemiological studies have suggested the primary target for these IgE antibodies to be a protein, Sm22.6 (also known as SmTAL1), which is characterized by a C-terminal region and two EF hand motifs, making them structurally similar to a very common group of clinical allergens known as the EF hand allergens [1,9]. Although its function is not fully delineated, this protein along with its orthologous counterparts, Sh22.6 (also known as ShTAL1) and Sj22.6 (also SjTAL1 in *S*. *japonicum*), are part of a family of recently-discovered 13 tegumental allergen-like proteins (TALs) associated with the vesicle-rich, syncytial outer layer of the schistosome [1,7,9,13,14].

Despite the observed structural similarity among the 13 TAL antigens, their expression profiles are varied throughout the schistosome life cycle, thus potentially influencing the varied specific antibody responses in the human host [15]. Recent transcriptomics studies have indicated the SmTAL1 to be abundant in the 24-hour schistosomulum and adult worm, but absent in the egg. Also, in treatment studies in *S*. *mansoni* endemic areas in Kenya and Uganda, levels of IgE to worm antigens SmTAL1 and SmTAL3 were observed to be high among participants and boosted when worms were disrupted *in vivo* by chemotherapy [1]. Although absent in the cercarium and 3-hour schistosomulum, SmTAL1, induces cross-reactive IgEs capable of recognizing SmTAL3 and SmTAL5 antigens present in these stages of the parasite [16]. Indeed, evidences from various studies suggest the SmTAL1 IgE response as a good marker for resistance to re-infection [1,16].

Given its structural homology to SmTAL1 (88% identical to SmTAL1) [17], it is anticipated that immune responses associated with ShTAL1 would be similar to those associated with SmTAL1 in a *S*. *haematobium*-endemic setting. In this intervention study, we sought to determine how ShTAL1 IgE levels would vary with certain risk factors at baseline (prior to PZQ treatment) and 7 weeks post-PZQ treatment; as a predictor for susceptibility or resistance among our selected participants. Assessment was also done against a humoral backdrop of IgG1, IgG4, and IgE responses to *S*. *haematobium*-specific adult worm antigen (ShAWA), and the *S*. *haematobium*-specific *Schistosoma* egg antigen (ShSEA). We also assessed the relationship between changes in ShTAL1 IgE and *S*. *haematobium* infection over the study period.

## Methodology

### Ethics statement

This work was approved by the Scientific and Technical Committee and ethical clearance granted by the Institutional Review Board all, of the Noguchi Memorial Institute for Medical Research with the certified protocol number, NMIMR-IRB CPN 005/11-12. Oral or written informed consent was obtained from opinion leaders, household heads and participating adults whilst parental consent was obtained for participants aged up to 17 years, as applicable. Oral or written informed assent was obtained from participants aged 12 to 17 years, as applicable.

### Study area

**Fig 1:**
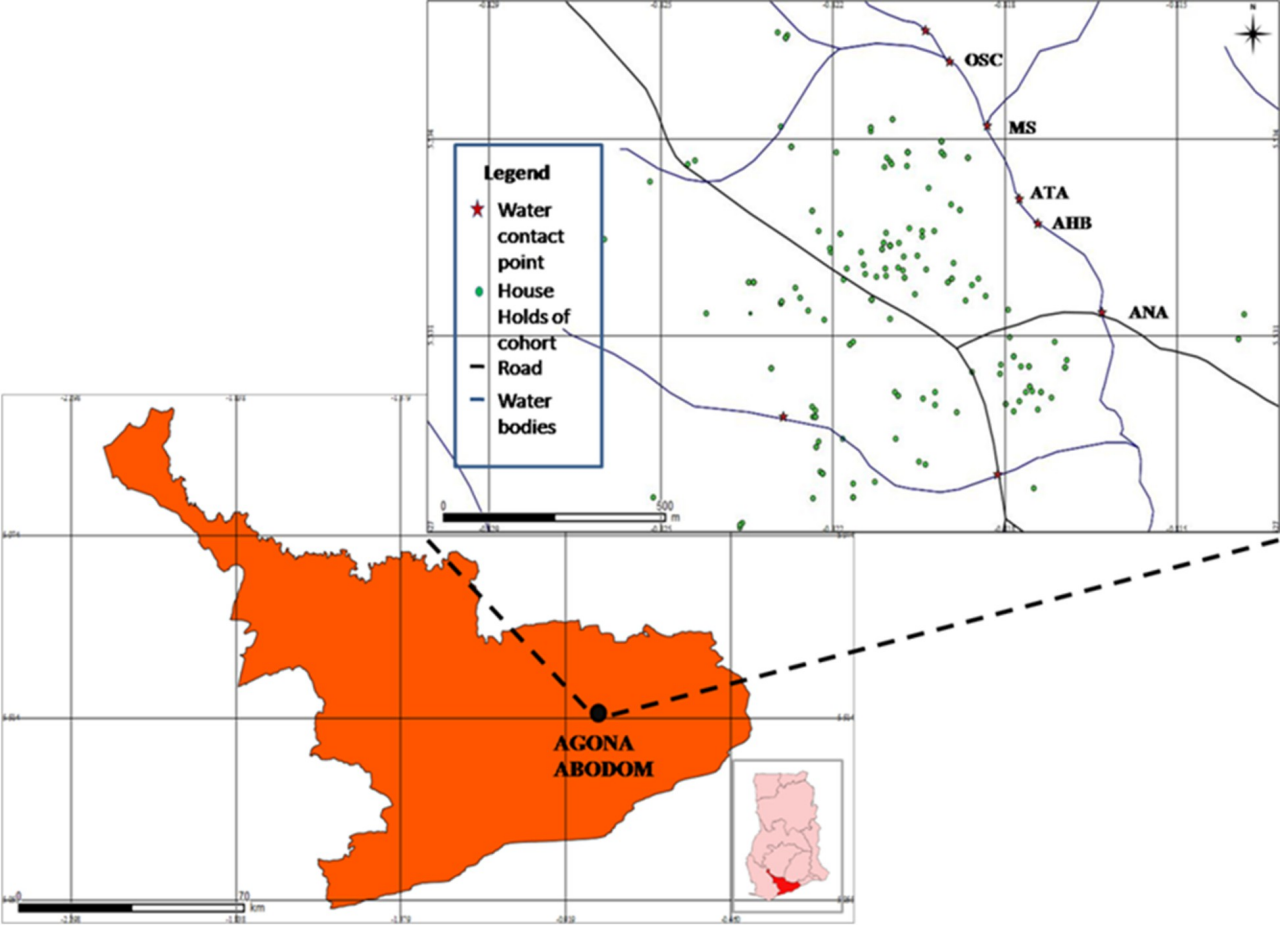
A map of the Central Region of Ghana with a zoom-in of the Agona Abodom community showing the spatial distribution of the water contact sites and houses of the 114 participants involved in the study. Map of the Central Region of Ghana is courtesy the Centre for Remote Sensing and Geographical Information Systems (CERSGIS), University of Ghana, Legon (2012). GPS data on households of study participants and water contact sites were plotted using the QGIS Valmiera 2.2 software (Essen, Germany, 2012). Legend of major water contact sites: OSC = Oworaa sika; MS = Mansowaa; ATA = Atifi Awombrew; AHB = Awombrew near High Blows Park; ANA = Anaafo Awombrew. Inset: Map of Ghana indicating the location of the Central Region. Figure was developed by Elias Asuming-Brempong.

Agona Abodom is a community in the Central Region of Ghana with approximately 8,200 residents, and lies within latitude 5.531321 N, and longitude -0.820068 W (Fig 1) [18]. The town lies within the moist tropical and semi-deciduous forest belt with rainy seasons occurring in May and September; and the dry season occurring between December and March. River tributaries surround Abodom, and are extensively accessed through 8 water contact sites by community members who depend on them for household and recreational purposes. The community has five basic and two Senior High schools. Farming is the principal occupation among adult residents, followed by skilled artisanal labour.

### Study design

This work was part of a larger, multi-centre study (TheSchistoVac, European Commission: HEALTH-2009-4.3.1–1) aimed at identifying putative candidates and developing a new generation vaccine for schistosomiasis. The study was conducted between 2010 and 2013 following ethical clearance as explained in the Ethics statement subsection.

Our sampling frame was shaped in part by a tarred road which ran through the Abodom township and split in a y-shaped form, dividing the community into three large quadrats (Fig 1). Households in these quadrats were enumerated and tracked with GPS devices (GARMIN LEGEND C Tracker, Kansas USA), and data initially entered into Microsoft Office Excel (MS Excel version 2007) from which 1,500 participants were randomly selected. A container was provided each participant following questionnaire administration, for the provision of single urine samples for screening. Of these, 840 samples were received, which were processed and examined by microscopy. Next, participants who were aged 5 to 56 years, and were not undergoing treatment for any ailments were further invited for blood draw. A total of 221 consented and provided urine and stool samples as well. Subsequent to the provision of requisite samples, participants were administered a single dose of PZQ (40mg/kg body weight; (Praziquantel B. P.-ERNEST CHEMIST LIMITED, Ghana) irrespective of their infection status. The direct observed treatment (DOT) method was employed, and each (especially young pupils) examined to ensure tablets were completely swallowed. Treatment for soil-transmitted helminths (STHs) was administered concurrently on subjects found positive, with a single dose of Albendazole (ALB, 400mg). Only 114 participants who had been treated with PZQ provided requisite samples for follow-up assessment 7 weeks later.

### Sample size determination and participant recruitment

**Fig 2:**
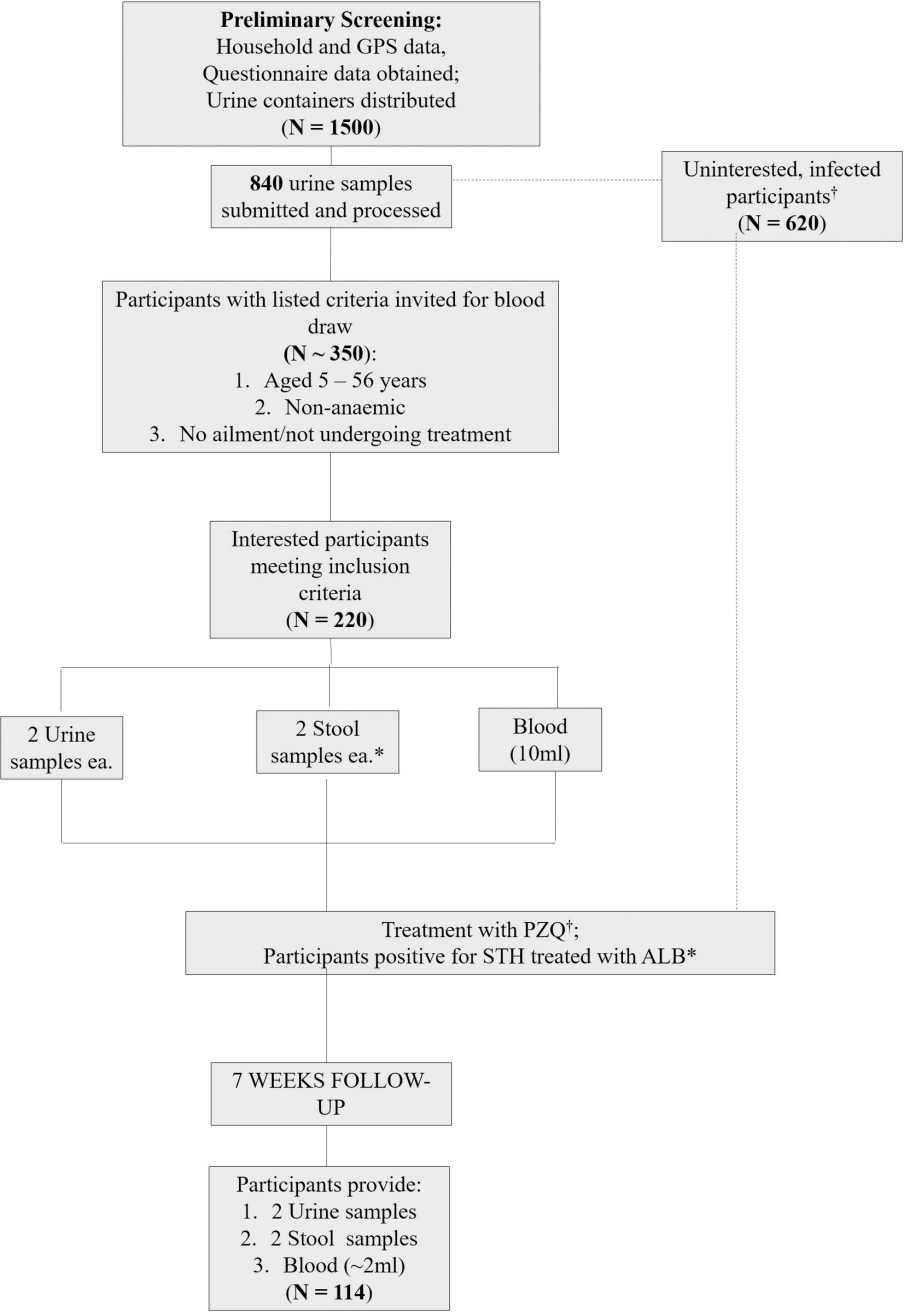
A flow chart illustrating the sequence of events/activities that characterized this intervention study. GPS = Global Positioning System; PZQ = Praziquantel; STH = Soil-transmitted helminths; ALB = Albendazole. [*] = Only participants who provided stool samples were assessed and treated for STH infections with ALB.

The representative number of participants for this endemic community was calculated to be 250, utilizing the formula: n = [2(Z_α_ + Z_β_)^2^σ^2^]/Δ^2^; where Z_α_ = 1.96 at a confidence interval of 95%; Z_β_ = 0.84 at 80% power; σ = a standard deviation of 0.5; and Δ = an estimated prevalence of 22.0% [19]. A fallout/non-response rate of 50% was anticipated, which when incorporated into calculations, engendered the estimated sample size indicated. We initially invited 1500 participants for screening, of whom 840 accepted by each presenting a single urine sample in containers provided. Subsequent to parasitological assessments, further invitation for blood draw was extended to participants in this selection who were non-anaemic (confirmed by qualified personnel using pallor); aged 5 to 56 years; and were not undergoing treatment for any ailments. A total of 220 participants subsequently presented themselves for blood draw. At follow-up, 114 of the 220 participants provided all requisite samples (i.e. urine, stool, and blood) and data (Fig 2). Pregnant women and nursing mothers were excluded from the study.

### Sample collection and processing

#### Stool

Each of the 220 participants provided 2 stool samples in wide-mouth screw cups on consecutive days. These were processed for microscopy using the Kato-Katz technique with the 41.70 mg card template [20,21]. Two slides were prepared per sample and examined for ova of soil-transmitted helminths (STH) and *S*. *mansoni*. Presence of ova was recorded and such samples designated positive for infection. The mean egg count from the two slides, designated as egg count per sample, was multiplied by a factor of 24 and expressed as eggs per gram (epg) stool.

#### Urine

Each participant also provided 2 urine samples (up to 50ml each) on consecutive days both at baseline and 7 weeks post-treatment. Each sample was processed by the filtration method as described elsewhere [22]. Two slides were prepared per sample and examined by microscopy for *S*. *haematobium* eggs, and a mean egg count determined. Intensity of infection was expressed as eggs per 10 ml of urine.

#### Blood

Approximately 2ml of EDTA blood sample collected per participant was each processed for haematology and antibody studies. Drops of blood from the syringe were used to prepare thick and thin films on microscope glass slides, which were Giemsa-stained and examined by microscopy to detect and estimate malaria parasitaemia. Parasite density per microliter was calculated from the number of parasites counted per 200 white blood cells (WBCs), multiplied by WBC count per microliter from haematological readings, and divided by 200 [23].

#### Antibody measurements by ELISA

An aliquot of 100ul of plasma from each participant was sent to the Department of Pathology, University of Cambridge (United Kingdom) for serological assessment. Antigen specific IgE, IgG1and IgG4 levels to *S*. *haematobium*-specific adult worm antigen (ShAWA), soluble egg antigen (ShSEA), and ShTAL1 were measured by ELISA as described previously [1]. Briefly, 384-well high-binding microplates (Greiner bio-one Ltd.) were washed with deionised water and coated with 15 μl of antigen in 0.1 M sodium bicarbonate pH 9.6 overnight at 4°C. Coating concentrations were 9.5, 9.1, and 2.4 μg/ml for ShAWA, ShSEA andShTAL1 respectively. The wells were washed 4 times with PBS containing 0.03% (v/v) Tween 20 using a BioTek ELx405 plate washer and blocked by incubating for 1 h with 1% (w/v) milk powder (Marvel) in PBS. To measure IgE, plasma was diluted with 10% (v/v) fetal calf serum (FCS) in ELISA buffer (PBS containing 0.1%(w/v) milk powder and 0.05% (v/v) Tween 20), at 1:20 dilution for ShTAL1 and 1:50 for ShAWA and ShSEA. To measure IgG1 and IgG4, plasma was diluted with 1%(v/v) FCS in ELISA buffer, at 1:200 dilution for ShTAL1 and 1:800 dilution for ShAWA and ShSEA. Following overnight incubation at 4°C, wells were washed again and incubated for 4 h with ELISA buffer containing 0.5 μg/ml biotinylated mouse anti-human IgE (Clone G7-26, BD Pharmingen, UK), 0.5mg/ml biotinylated IgG1 (Clone G17-1, BD Pharmingen) or 0.5 μg/ml biotinylated mouse anti-human IgG4 (Clone G17-4, BD Pharmingen). After washing, wells were incubated for 1 h with streptavidin/biotinylated–horse radish peroxide complex (Mast Group Ltd, UK), diluted at 1/3000 in ELISA buffer and then washed again. The assay was developed with 68 μl o-phenylenediamine substrate solution (Sigma-Aldrich, USA) and stopped with 17 μl of 2 M sulphuric acid as required. A standard curve was generated by coating a serial dilution of human myeloma proteins, IgE (Merck Millipore, Germany), IgG1 (Sigma-Aldrich, USA) or IgG4 (Sigma-Aldrich, USA) as appropriate, and plasma samples from 26 uninfected European donors were included in each assay. The plates were read using a Powerwave HT reader (BioTek Instruments Inc., USA) at a test wavelength of 490 nm and a reference wavelength of 630 nm. The OD values from the myeloma data were used by the ELISA reader software to generate standard curves using 5-parameter logistic regression. Cut-off values to distinguish responders to an antigen of interest from non-responders were determined using the mean and standard deviation values calculated from the negative controls, specifically:

‘threshold’ value = mean + 3x (Standard Deviation (SD). The list of threshold/cut-off values for each antigen is presented in Table A in S1 Text.

#### Treatment with Praziquantel

The height of each participant was measured using the WHO PZQ dose pole based on which the appropriate number of tablets were given, along with an adequate amount of water for swallowing [24]. The mouths of each participant was examined to ensure the medication was completely ingested, and the individuals further observed for any transient reactions that could occur.

### Data analyses

The main hypothesis tested in this study was the influence of Praziquantel (PZQ) treatment on ShTAL1 IgE antibody levels, using the following null and alternative hypotheses:

*H*_*0*_ = There is no significant change in ShTAL1 IgE levels of participants following PZQ treatment; and

*H*_*1*_ = There is significant change in ShTAL1 IgE levels of participants subsequent to treatment with PZQ.

The statistical software used in data analyses and graphical illustrations were SPSS version 20.0 (IBM Corp, New York, USA), Microsoft Office Excel 2010 (Microsoft Corporation, USA), and Graph Pad Prism Version 7.0 (Graph Pad Software, La Jolla CA, USA) respectively. Interval parameters which were non-parametric were converted to ordinal and nominal forms prior to analyses, and corresponding tests employed. Where appropriate, the Chi-square, Mann-Whitney U, and Kruskal-Wallis tests were used in identifying differences between independent groups at a particular time point, whiles the Related Samples McNemar and Wilkoxon Matched-Pairs Signed-Ranks tests were used in detecting variations for a particular group over time. In testing the second hypothesis, the generalized estimating equation (GEE) was employed in which treatment was used as the within-participant variable. A product term was then fitted in the univariate model to examine the influence of *S. haematobium* infection and PZQ treatment on ShTAL1-IgE positivity. Other *a priori* factors tested in the univariate model included age, gender, soil-transmitted helminth (STH) infection status, and *Plasmodium spp*. infection status. Significant associations/interactions in the univariate model were then fitted in the multivariate analysis to adjust for confounders. Associations with p-values less than 0.05 were deemed significant.

## Results

### Characteristics of study participants

A total of 840 participants were initially screened, out of which 220 (26.31%) participants were recruited, and 114 were successfully followed up on at 7 weeks post-PZQ treatment (Fig 2). The 114 comprised 66 (57.90%) males and 48 (42.10%) females (p = 0.09) aged 6 to 55 (median: 12) years. Of the 114, 39 (34.20%) were aged 6 to 11 years, 42 (36.80%) 12 to 14 years, and 33 (28.90%) ≥ 15 years (p = 0.58) (Table 1).

**Table 1:** Characteristics of Study Cohort.

Factor	Baseline (N = 114)	Follow-Up (7 weeks Post-Treatment (N = 114))	p-value^§^
	n (%)	n (%)	
Gender			
Male	66 (57.90)	-	0.09
Female	48 (42.10)	-	
Age in years: Median (range)	12 (6–55)	-	-
Age group in years			
6–11	39 (34.20)	-	0.58
12–14	42 (36.80)	-	
≥15	33 (28.90)	-	
*S*. *haematobium* infection^§^			
Positive	26 (22.80)	4 (3.50)	**<0.001**
STH Infection status^§§^			
Positive	8(7.0)	n(0.90)	**0.02**
*P*. *falciparum* Parasitaemia^§^			
Positive	47 (41.22)	43 (37.72)	0.461
Negative	67 (58.77)	71 (62.28)	

**§** The McNemar test was used to determine level of significance between pre-and post-treatment data. Significant values are in boldface.

**§§** STH refers to soil-transmitted helminths, namely *Ascaris lumbircoides*, and *Trichuris trichiura*.

### Prevalence and intensity of parasitic infections; and assessment of degree of exposure to infested water

In Agona Abodom, *S*. *haematobium* is endemic, whilst *S*. *mansoni* is completely absent. Prevalence of *S*. *haematobium* infection at baseline was 22.80% (26/114), and 3.50% (4/114) at 7 weeks post-PZQ treatment (henceforth denoted as Follow-up) (p < 0.001). For STH (*Ascaris lumbricoides* and *Trichuris trichiura*), baseline prevalence was 7.00% (8/114), and 0.90% (1/114) at Follow-up (i.e. 7 weeks post-PZQ treatment; p = 0.02). Baseline prevalence of *Plasmodium falciparum* parasitaemia was 41.22% (47/114), and reduced to 37.72% (43/114) at Follow-up (p = 0.38) (Table 1).

Overall geometric mean (GM) intensity for *S*. *haematobium* infection at baseline was 22.77; and 1.41 eggs/10ml urine at Follow-up (p < 0.001). This translated into a total egg reduction rate (ERR) of 99.87%. Similarly, the GM intensity for *A*. *lumbricoides* was 3,222.42 eggs per gram of stool (epg); and no eggs were detectable at Follow-up (p < 0.001). For *T*. *trichiura* the GM infection intensity was 24 epg at both baseline and Follow-up. Geometric mean for *P*. *falciparum* parasitaemia also decreased from 6.43 at baseline to 0.21 at Follow-up (p < 0.001) (Table 2).

**Table 2:** Descriptive summary of interval parameters of interest.

**Parameter**	**Pre-treatment**	**Post-treatment**	**p-value**
	Parasite infection intensities: GM (range)		
*S*. *haematobium*^1^	22.77 (1–1294)	1.41 (1–2)	**<0.001**
*P*. *falciparum* parasitaemia^2^	6.43 (1–174)	0.21 (0.02–3.54)	**0.001**
*A*. *lumbricoides* infection intensity^3^	3222.42 (0–72000)	0 (0)	**0.012**
*T*. *trichiura* infection intensity^4^	24 (24)	24 (24)	0.66
	Haematology: Mean (95% CI)		
Red blood cells^5^	4.57 (3.38–5.98)	4.57 (3.31–5.98)	0.68
Haemoglobin ^6^	11.91 (9.80–16.70)	11.70 (3.40–10.70)	**0.004**
White blood cells^7^	6.89 (3.00–13.00)	6.66 (3.40–10.70)	0.22
Lymphocytes^8^	3.53 (1.30–6.80)	3.58 (0.20–6.20)	0.41
Neutrophils^9^	2.71 (1.00–7.50)	2.55 (0.80–5.80)	0.20

^1^ = eggs/10ml urine

^2^ = counts per 200 wbc

^3,4^ = eggs/gram stool

^5^ = x10^6^/μL

^6^ = grams/dL

^7^ = x10^3^/μL

^8^ = x10^3^/μL

^9^ = x10^3^/μL. The Wilkoxon Matched Pairs Sign-rank test was used in determining the p-value between the time-points for each of the parameters.

Assessment of questionnaire data on water contact information indicated firstly that 95 (83.33%) of the 114 participants relied on the stream/water body as their main source of water. Furthermore, 92 (80.70%) visited the water body regularly, with frequencies ranging between ‘once a month’ to ‘two or more times a day’. Of the 26 *S*. *haematobium*-positive cases recorded among our participants, a little over half (i.e. 15; 57.70%) indicated they visited the water body at least twice a week (Table D in S1 Text). We also identified 8 water contact sites in the community, and extracted *Bulinus sp*. snails from five.

### Association of *S*. *haematobium* infection with age and gender

**Fig 3:**
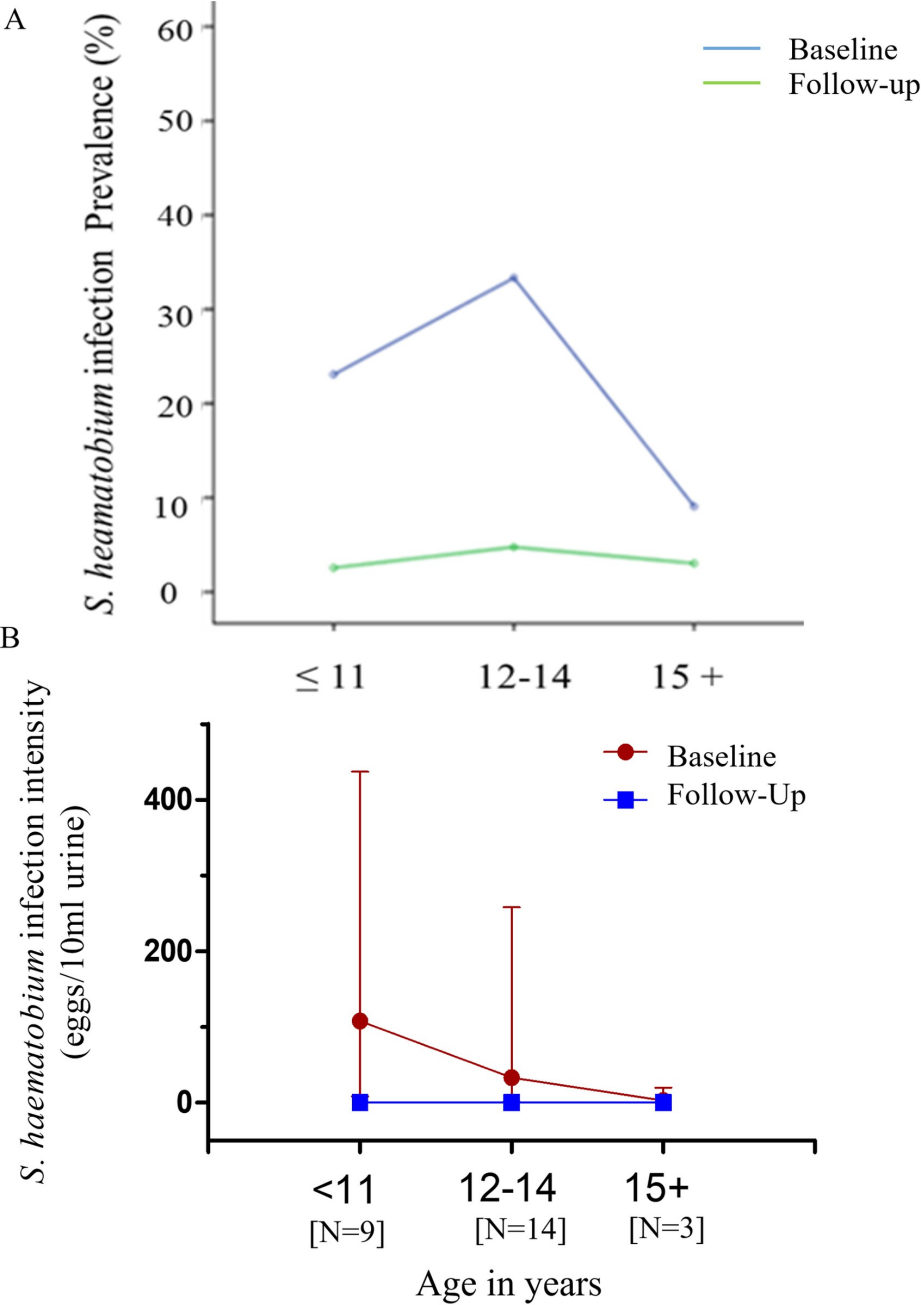
(A) Percent prevalence of *S*. *haematobium* infection among infected participants (N = 26) at baseline and follow-up (7 weeks post-PZQ treatment) for the study cohort. (B) Geometric mean intensity of *S*. *haematobium* infection at baseline and follow-up stratified by age in study cohort. Error bars denote 95% confidence intervals.

Baseline *S*. *haematobium* infection prevalence was highest among infected participants aged 12–14 years (N = 14), followed by that for participants aged 11 years or less (N = 9), and for participants aged 15 years and above (N = 3). Infection prevalence at Follow-up was almost zero for all groups. Baseline GM *Schistosoma haematobium* infection intensity however decreased with increasing age, with participants aged 11 years or less exhibiting the highest intensities, followed by participants in the 12-14-, and in the 15-years and above age groups exhibiting progressively lower infection intensities. Follow-up GM infection intensities reduced to near-zero levels for all groups (Fig 3A).

Although not statistically significant, higher GM infection intensity levels were reported among females, whilst higher prevalence levels were reported among males at baseline. Infection intensity and prevalence levels reduced significantly however, in males and females at follow-up (Fig A in S1 Text).

### Antibody response profiles

The overall percent frequencies of positive responders at baseline for *S*. *haematobium*-specific AWAIgG1 (ShAWAIgG1), ShAWAIgG4, ShAWAIgE, ShSEAIgE, ShTAL1IgG1, ShTAL1IgG4, and ShTAL1IgE, increased 7 weeks following PZQ treatment (Fig 3A). This trend was similarly observed when assessment was restricted to *S*. *haematobium*-infected participants (i.e. N = 26; Fig 3B). Furthermore, higher ShTAL1 IgE percent frequency was recorded among males than females at both baseline (p < 0.01) and 7 weeks post-PZQ treatment (p<0.01) (Fig B in S1 Text).

**Fig 4:**
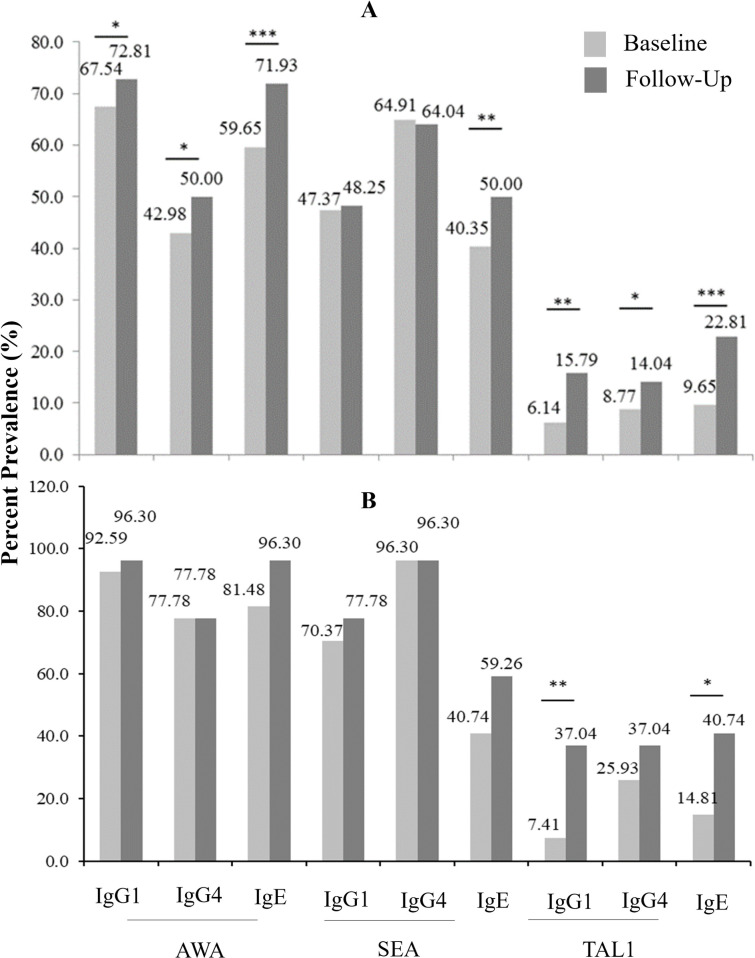
Percent frequency of antibody responses to *S*. *haematobium*-specific antigens at baseline and 7 weeks post-Praziquantel (PZQ) treatment. (A) Overall percent frequency of positive antibody responders to the *S*. *haematobium* antigens under scrutiny both at baseline and follow-up. (B) Percent frequency of positive antibody responses among treated S. *haematobium*-infected participants (N = 26) to *S*. *haem*a*tobium* antigens under scrutiny both at baseline and follow-up. The Related Samples McNemar Test was employed in determining p-values. * is indicative of p-values < 0.05, whilst ** is indicative of p-values <0.01, and *** is indicative of p-values < 0.001. ShTAL1 = *S*. *haematobium* (*Sh*)-specific tegumental allergen-like protein 1; ShAWA = *S*. *haematobium* (*Sh*)-specific adult worm antigen; ShSEA = *Sh*-specific Soluble Egg Antigen.

Median titres of ShAWA-, and ShTAL1-specific IgG1 and IgG4 antibodies generally increased at follow-up for the study cohort (N = 114), except for ShSEA-specific IgG1 and IgG4 levels (Fig 4). Overall ShAWA-, ShSEA-, and ShTAL1-specific IgE levels also increased at follow-up (p < 0.001 with respect to all antigens (Fig C in S1 Text). Restricting assessments to participants infected with *S*. *haematobium* at baseline (N = 26), presented with similar outcomes, again with no significant change in ShSEA-specific IgG1 and IgG4 antibody levels at follow-up. Increased levels of ShAWA-, ShSEA-, and ShTAL1-specific IgE were also observed at Follow-up (Fig D in S1 Text).

### Age-associated *S*. *haematobium*-specific antibody levels at baseline and follow-up for *S*. *haematobium*-infected participants (N = 26)

**Fig 5:**
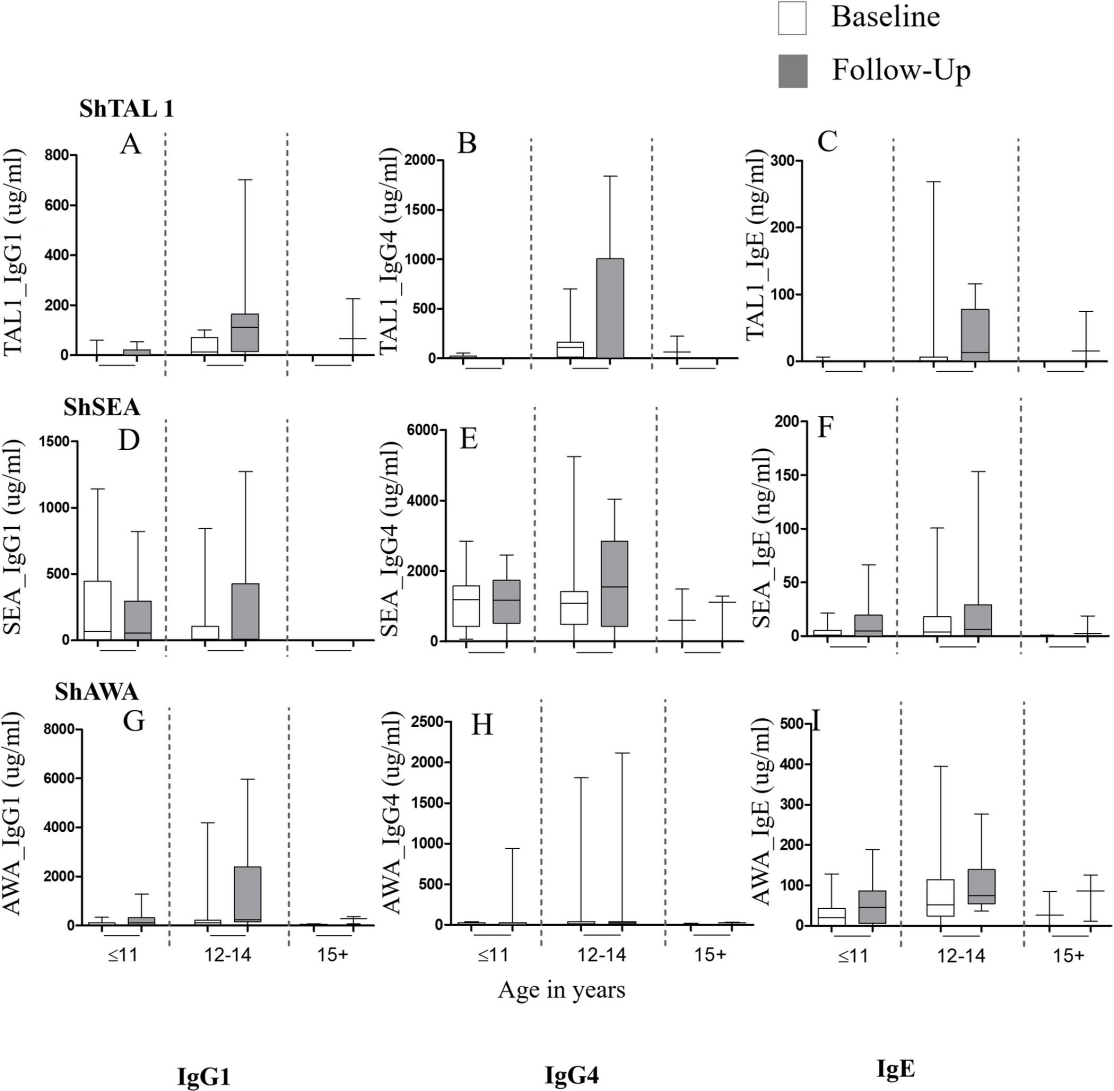
Association of median titres of *S*. *haematobium*-specific antibodies with age. Association of median titres of *S*. *haematobium*-specific antibodies (A–I) with age at baseline and Follow-up among participants infected with *S*. *haematobium* at baseline (N = 26). Horizontal lines within the boxes (or those bisecting whiskers) denote the median levels. The lower horizontal border of the box represents the 25^th^ percentile, whilst the upper boarder denotes the 75^th^ percentile. The upper and lower whiskers are set at the 95^th^ and 5^th^ percentiles respectively. The Wilkoxon Matched-Pairs Signed-Ranks test was employed in determining p-values.

**Fig 6:**
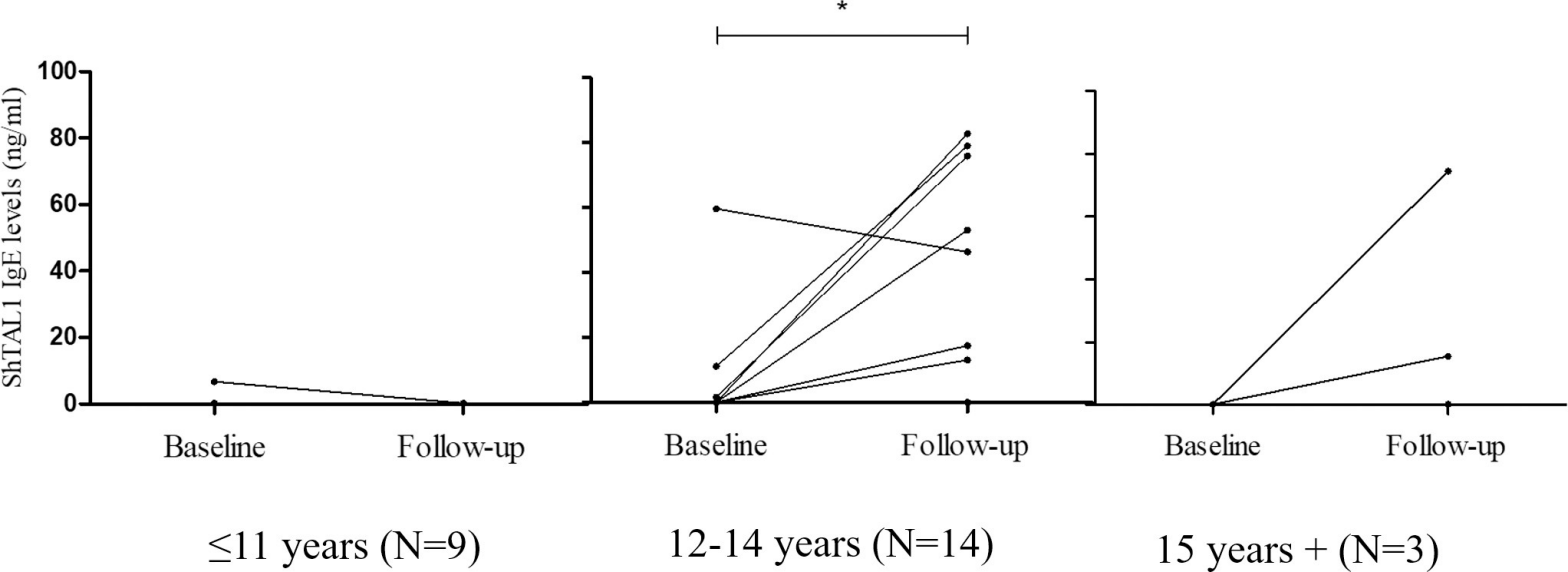
Before-after line graphs of participant ShTAL-IgE titres stratified by age groups. Baseline and post-treatment comparisons within each age groups was conducted using the Wilcoxon paired sign-rank test. * denotes p-values less than 0.05.

For *S*. *haematobium*-infected participants (N = 26), ShTAL1-specific antibodies presented with varied age-associated trends at baseline, and at Follow-up. ShTAL-specific IgG1 and IgG4 antibodies exhibited similar trends at baseline, whereby median titres increased from almost nil for participants aged 11 years or less, and peaked among participants aged 12–14 years. ShTAL1-IgG1 and–IgG4 titres declined with further increase in age, with median–IgG1 titres almost 0ug/ml for participants aged 15 years and above (Fig 5). Median ShTAL1-IgE levels were however hardly detectible for all age groups at baseline. At Follow-up, median ShTAL1-IgE levels increased with increasing age groups. Median ShTAL1-IgG1 levels also increased considerably, peaking for participants aged 12–14 years, and decreasing slightly with further increase in age. Median ShTAL1-IgG4 levels were however hardly detectible for all age groups (except for the 12-14-year age group) at Follow-up (Fig 5). Age-associated before-after plots developed with regard to ShTAL1-IgE levels indicated hardly any detection of ShTAL1-IgE among our participants aged 11 years or younger (N = 8) at both baseline and Follow-up; whilst among our participants aged 12 to 14 years (N = 12) and 15 years and older (N = 3), ShTAL1IgE levels increased at Follow-up from hardly detectible levels at baseline (Fig 6).

Baseline ShSEA- and ShAWA-specific IgE titres were observed with age-associated trends similar to those observed for ShTAL1-IgG4. Thus, median IgE titres peaked among participants aged 12–14 years, and were low both among participants aged 11 years or less, and participants aged 15 years or older (Fig 5). At Follow-up, median ShSEA-IgE levels remained low, declining with increasing age, whilst ShAWA-IgE titres exhibited trends similar to what was observed for ShTAL1-IgE at Follow-up. Median ShSEAIgG4 titres at baseline were similar among participants aged up to 14 years, and decreased with further increase in age (Fig 5). Baseline ShAWA-IgG4 median titres were highest for participants aged 12–14 years, decreasing with further increase in age. Participants aged 11 years or less presented with the lowest titres. At Follow-up however, both ShSEA- and ShAWA- IgG4 median titres peaked for participants aged 12–14 years, and decreased with further increase in age (Fig 5).

### Association of risk factors with ShTAL1 IgE positivity

Using ShTAL1 IgE response as the outcome variable in the crude analysis, the influence of gender, age, *P*. *falciparum*, and STH infections were tested. Additionally, the combined effect of treatment and *S*. *haematobium* infection status on ShTAL1-IgE positivity was also assessed. Each of these factors were first assessed in the univariate model, and subsequently inputted together in a multivariate analysis to adjust for confounders. In the crude analysis, being *S*. *haematobium—*positive (crude odds ratio (cOR) = 13.33; 95%C.I. = 1.58–112.01; p = 0.017), or -negative (cOR = 3.81; 95% C.I. = 1.84–7.89; p < 0.001) at Follow-up increased a participant’s likelihood for ShTAL1-IgE positivity; along with *P*. *falciparum* infection positivity (cOR = 4.66, 95% CI = 1.69–12.79; p = 0.003). The risk of odds for ShTAL1 IgE positivity increased with increasing age, with the ≥ 15-year age group having the highest predictive value (cOR = 14.26, 95% CI = 3.10–65.51; p = 0.001), followed by the 12-14-year age group (cOR = 9.64, 95% CI = 2.09–44.51; p = 0.004). In the adjusted model, age [(12–14 years: aOR = 22.34, 95% CI = 2.77–180.14; p = 0.004), (≥15 years: aOR = 51.82, 95% CI = 6.44–417.17; p < 0.001)] and being *S*. *haematobium*-negative at Follow-up (aOR = 5.38, 95% CI = 2.48–11.64; p < 0.001) remained significantly associated with ShTAL1 IgE positivity (Table 3).

**Table 3:** Factors associated with changes in ShTAL1 IgE response over the study period.

Factor	ShTAL1-specific IgE (Responder vs. non-responder)			
	cOR (95% CI)	P-value of interaction	aOR (95% CI)	Wald’s P-value
*S*.*haematobium*. Infection * Treatment				
*S*. *h*. positive * Baseline	1.74 (0.40–7.49)	0.458	-	-
*S*. *h*. negative * Baseline	1.00 (0.00)	-	-	-
*S*. *h*. positive * Follow-up	**13.33 (1.58–112.01)**	**0.017**	6.16 (0.71–53.59)	0.099
*S*. *h*. negative * Follow-up	**3.81 (1.84–7.89)**	**<0.001**	**5.38 (2.48–11.64)**	**<0.001**
STH Infection status				
Negative	1	-	1	-
Positive	1.00 (0.00)	1.00	3.94 (0.57–27.14)	0.16
Age (years)				
6–11	1	-	1	-
12–14	**9.64 (2.09–44.51)**	**0.004**	**22.34 (2.77–180.14)**	**0.004**
≥ 15	**14.26 (3.10–65.51)**	**0.001**	**51.82 (6.44–417.17)**	**<0.001**
Gender				
Male	1	-	1	
Female	0.53 (0.22–1.28)	0.16	0.51 (0.17–1.52)	0.225
*P*. *falciparum* infection status				
Negative	1	-	1	-
Positive	**4.66 (1.69–12.79)**	**0.003**	2.14 (0.98–4.69)	0.058

The Generalized Estimating Equation was used in determining the association of factors with ShTAL1 IgE response frequencies at baseline and at Follow-up (i.e. 7 weeks post-PZQ treatment). A product term was employed to assess the combined effect of *S*. *haematobium* infection status and treatment on ShTAL1 IgE positivity. cOR = crude Odds Ratio; aOR = adjusted Odds ratio; STH = soil-transmitted helminths comprising *Ascaris lumbircoides*, and *Trichuris trichiura*; ShTAL1 = *S*. *haematobium*-specific tegumental allergen-like protein 1. Significant values are in boldface.

## Discussion

In this community-based, intervention study, we present data that suggest the association of ShTAL1-IgE responses with a reduced risk to re-infection 7 weeks after PZQ treatment (Follow-up), as has been similarly shown for a cohort of Gabonese schoolchildren [25], and for IgE responses to its orthologous *Schistosoma mansoni* antigen, SmTAL1.

First, the overall percent frequency of ShTAL1-IgE response increased significantly for our study cohort at Follow-up; and this was similarly observed among *S*. *haematobium*-infected participants (N = 26). Buttressed by outcomes in our univariate analysis which indicated increased odds for positive ShTAL-IgE response at Follow-up regardless of infection status, it is apparent that, increased ShTAL1-IgE response is strongly associated with PZQ treatment. Also, in the multivariate analysis, being *S*. *haematobium*-negative after treatment remained significantly associated with positive ShTAL1-IgE response after adjusting for confounders. These observations compare with findings reported in Uganda, Kenya, and Gabon [11,12,26] which showed increased ShTAL1-, and SmTAL1-IgE responses following PZQ treatment. From earlier immune localization studies conducted on the 22.6 kDa tegumental antigens of *S*. *mansoni* (Sm22.6) and *S*. *japonicum* (Sj22.6), it was realized that neither the Sm22.6 nor the Sj22.6 antigens were exposed on the adult worm surfaces [27,28]), suggesting that -specific IgE recognition was most probable following the disruption and/or damage of the outer syncytial layer of the adult worm via chemotherapy or natural worm death over time. This likely explains the increased frequencies in ShTAL1 IgE response at Follow-up, given the striking structural homology between Sm22.6 and Sh22.6 [25].

Second, we observed among *S*. *haematobium*-infected participants (N = 26), inverse trends at both time points, of age-associated median ShTAL1-IgE and–IgG4 titres. While minimal median ShTAL1-IgE titres were observed for all age groups at baseline, median ShTAL1-IgG4 increased concurrently from minimal levels among participants 11 years or less, peaking among participants aged 12 to 14 years, and decreasing slightly among participants 15 years or older. At Follow-up, the inverse was apparent, whereby minimal median ShTAL1-IgG4 titres were realized for all age groups, whilst median ShTAL1-IgE levels increased from minimum among subjects aged up to 11 years, and peaked among participants aged 15 years or older. Indeed, in our multivariate analysis, likelihood for ShTAL1-IgE positivity increased significantly with age among participants aged 12 years or older for this cohort, and this remained significant after adjusting for confounders. Our observations with regard to age-associated median ShTAL1-IgE trends at post-treatment corroborate proposals made by Fitzsimmons and colleagues [25], as well as by de Moira and team [12], about age being a positive predictor of developing resistance, as it underscores the likely length and extent of previous sensitizations to key *S*. *haematobium* antigens among participants who have lived all/a large part of their lives in an endemic setting. Additionally, given the lifespan of the *Schistosoma* adult worm, which is up to 7 years in a human host [12], participants 11 years or younger are likely to have had less exposure to the TAL1 antigen due at least, to natural worm death or chemotherapy, as compared to participants 12 years and older, as our data indicates. Evidence from immuno-epidemiological studies conducted among preschool-aged children (PSAC) in *S*. *mansoni*-endemic communities in Uganda, and among indigenes (aged 5 to 48 years) in Lambarene, Gabon, indicate minimal SmTAL1-IgE responsiveness among the PSAC in Uganda 7 weeks after treatment with PZQ [12]; and comparatively much less ShTAL1–IgE responses among recruited children, as compared to recruited adults, in the Gabonese cohort 5 weeks post-PZQ treatment [17].

The clear, inverse trends observed among our treated participants between age-stratified median ShTAL1-IgE and–IgG4levels in this study most likely indicate a balance between the IgE and IgG4 antibodies that reflect growing resistance to *S*. *haematobium* re-infection in our cohort, as proposed by a number of previous studies [15,26,29,30]. Indeed, the outcomes we observed are in agreement with the theory which suggests elevated levels of IgE to be associated with immunity to re-infection (present often during acute infections and upon treatment); whilst IgG4 is said to have a blocking effect through competition with IgE for same antigenic epitopes, and is often an indication of an active regulatory immune response mediated by increased Interleukin (IL)-10-producing regulatory T-cell activity [31]. It would therefore be anticipated that resistance to re-infection with *S*. *haematobium* may be characterized by increased levels of ShTAL1-IgE and much lesser levels of ShTAL1-IgG4 among participants showing no signs of infection after treatment–or a balance between ShTAL1-IgE and–IgG4 indicative of a more dominant–IgE response. However, the associated patterns defining such a balance appear to vary between studies. While de Moira and team [12], in a study assessing IgE, IgG1,and IgG4 responses to *Schistosoma mansoni* and hookworm antigens in Ugandan school children co-infected with *S*. *mansoni* and hookworm, observed 8 weeks after PZQ treatment, much more elevated SmTAL1-IgE levels as compared to SmTAL1-IgG4 levels for higher age groups (10 to 14 years); Fitzsimmons and colleagues [1], in a study in a fishing community (Musoli) in Uganda involving males aged 7 to 76, observed increases in SmTAL1-IgE and–IgG4 post-treatment with PZQ that were highly correlated in both magnitude and response prevalences. In another study by de Moira et al., [15] in another fishing village (Booma) in Uganda, they observed a reduced odds for re-infection among participants who were SmTAL1-IgE-positive at 12 months post-PZQ treatment, and the converse among participants who were SmTAL1-IgG4-positive. Thus, although outcomes in these studies agree with our findings, in terms of age-associated post-treatment TAL1-IgE levels, associated TAL1-IgG4 responses may require further studies incorporating different geographical settings of endemicity among others.

Interestingly, we observed among our participants who remained infected after treatment (N = 4), a positive relationship between ShTAL1-IgE and–IgG4 levels, whereby titres of both antibodies increased sharply at Follow-up, with much higher ShTAL1-IgG4 titres recorded. Again, this observation falls well within the hypothesis that elevated levels of IgG4 are most likely associated with susceptibility to schistosomiasis re-infection [25,32,33], one which could not be tested in this work. For these participants, incomplete clearance of the parasite during treatment; or the presence of residual infections, may possibly explain why they remained infected.

This study however is not without important limitations. One of such is the design, which would have facilitated clear stratification of our participants into *S*. *haematobium*-susceptible, or -resistant, groups, had a 12-month post-treatment assessment been carried out. Given that, it is worth noting that evidence from previous separate studies carried out by de Moira and team in Booma [15] and Bwondha [12], in eastern and north-western Uganda, suggest that immune responses measured 7 weeks post-PZQ treatment are likely to be more closely related to re-infection at 12 months. Secondly, a PZQ mass chemotherapy exercise which was undertaken by the local health authorities on pupils attending public schools in Abodom nearly 6 weeks prior to the commencement of our work may have influenced outcomes herein presented. This may be the cause for the higher number of positive antibody responders to ShAWA, ShSEA, and ShTAL1 antigens observed, as compared to the number of *S*. *haematobium*-positive cases at baseline. Unfortunately, accurate records of pupils who participated in the exercise were not kept; and given that pupils aged 6 to 17 years comprise circa 90% of our cohort, their involvement in this study as well is highly likely. Another important limitation was the number of urine and stool samples collected for parasitological assessment at each time point. Given the moderate prevalence of schistosomiasis in the Abodom community (~23%), and the low sensitivity of the filtration and Kato-Katz techniques, a minimum of 3 urine and stool samples per recruited participant on consecutive days had been initially determined, as this appeared to be standard practice in related studies [12,16,34,35]. Indeed we began collection of samples on three consecutive days per recruited participant, but waning participant cooperation and the increasing number of refusals forced us to modify our sample collection strategy. For each stool and urine sample collected however, we prepared 2 slides for examination by microscopy. We also conducted with the aid of experienced technicians, quality assurance checks on every tenth slide examined. A fourth limitation of this work was the final number of recruited participants who fully cooperated throughout the study. We were informed by resident health workers of superstitious beliefs indigenes of Abodom had concerning urine, stool, and blood. To mitigate this challenge, a number of durbars were organized prior to work commencement: the first involved the entire community comprising inhabitants, the chief, and the opinion leaders; whilst the second involved a motion picture presentation on schistosomiasis. Subsequent community durbars aimed at updating community members on various phases of the study were also held. Despite the combined extensive efforts of resident health workers and our team at engaging indigenes both individually and communally, fallout rates were still high, affecting the power of our study.

In summary, we provide evidence from this community-based intervention study indicating response patterns of ShTAL1-IgE that are similar at 7 weeks post-PZQ treatment to SmTAL1- and ShTAL1-IgE responses reported elsewhere [12,15]. Such responses are characterized by age-associated increases, which in our case, is suggestive of increased protection against *S*. *haematobium* re-infection. Having clear indicators to determine a participant’s degree of susceptibility to schistosomiasis re-infection will prove essential in the coming years as the World Health Organization with its donor partners intensify commitment to eliminating neglected tropical diseases (NTDs) as a Public Health Problem. In the very least such clear stratifications among indigenes in endemic areas could enable for the strategic disbursement of much-needed medications and resources to persons and beneficiaries who most need them. More importantly though, intensified treatment activities must be coupled with increased investment into providing certain amenities in such areas of endemicity (such as potable water). This will ensure that gains made through mass drug administration programmes are not reversed.

## Supporting information

S1 Text **Table A:** List of threshold/cut-off values for IgG1, IgG4, and IgE responses to the *S*. *haematobium* antigens studied. **Table B:** Number of baseline responders to the *S*. *haematobium* antigens as compared to number of participants positive of *S*. *haematobium* infection at baseline. **Table C:** Information details on the 4 individuals who remained infected at Follow-Up (7 weeks post-PZQ treatment). **Table D:** Frequency of contact with, and extent of use of water body in the Abodom community. **Fig A:** Associations of Participant gender with *S. haematobium* (A) infection intensity and (B) Prevalence. The Wilcoxon paired matched sum sign rank test (A), and the Related Samples McNemar Test (B) were used to assess for significant differences in infection levels/prevalences over time for each group. The Mann Whitney U (A) and the χ^2^ tests were utilised to assess for significcant differences between groups at a particular time point. * is indicative of p-values < 0.05; *** is indicative of p-values < 0.001. **Fig B:** Associations of Participant gender with ShTAL1-IgE (A) percent response frequency and (B) levels at baseline and 7 weeks post-PZQ treatment. The Related Samples McNemar Test (A), and the Wilcoxon paired matched sum sign rank test (B) were used to assess for significant differences in infection levels/prevalences over time for each group. The X^2^ (A) and the Mann Whitney U tests were utilised to assess for significant differences between groups at a particular time point. ** is indicative of p-values < 0.01; and *** is indicative of p-values < 0.001 **Fig C: Antibody levels to *S*. *haematobium*-specific antigens at baseline and 7 weeks post-Praziquantel (PZQ) treatment**. (A) Box-and-whisker plots indicate differences in IgG1 and IgG4 titres at pre- and post-treatment to ShTAL1, ShAWA, and ShSEA. (B) Differences in IgE titres at baseline and 7 weeks post-treatment to *S*. *haematobium*-specific TAL1, AWA, and SEA. Lines within the boxes indicate median responses, whiles upper and lower whiskers are set at the 95^th^ and 5^th^ percentiles, respectively. The Wilkoxon Matched-Pairs Signed-Ranks test was employed in determining p-values. ** is indicative of p-values < 0.01, whilst *** is indicative of p-values <0.001. ShTAL1 = *S*. *haematobium* (*Sh*)-specific tegumental allergen-like protein 1; ShAWA = *S*. *haematobium* (*Sh*)-specific adult worm antigen; ShSEA = *Sh*-specific schistosome egg antigen. **Fig D: Antibody levels to *S*. *haematobium*-specific antigens at baseline and Follow-up (7 weeks post treatment with PZQ) for *S*. *haematobium*-infected participants who were egg-negative after PZQ treatment (N = 26):** (A) Box-and-whisker plots indicate differences in IgG1 and IgG4 titres at pre- and post-treatment to ShTAL1, ShAWA, and ShSEA. (B) Differences in IgE titres at baseline and 7 weeks post-treatment to *S*. *haematobium*-specific TAL1, AWA, and SEA. Lines within the boxes indicate median responses, whiles upper and lower whiskers are set at the 95^th^ and 5^th^ percentiles respectively. The Wilkoxon Matched-Pairs Signed-Ranks test was employed in determining p-values. * is indicative of p-values < 0.05, whilst ** is indicative of p-values <0.01. ShTAL1 = *S*. *haematobium* (*Sh*)-specific tegumental allergen-like protein 1; ShAWA = *S*. *haematobium* (*Sh*)-specific adult worm antigen; ShSEA = *Sh*-specific schistosome egg antigen. **Fig E:** Percent prevalences of IgG1, IgG4, and IgE antibody responses to *S*. *haematobium*-specific AWA, SEA, and TAL1 stratified by age at pre- and post- PZQ treatment. AWA = adult worm antigen; SEA = soluble egg antigen; TAL1 = tegumental allergen-like protein 1.
(DOCX)

S1 Data Manuscript Dataset in SPSS format. (SAV)
