# Supplementary material for: Increased ShTAL1 IgE responses post-Praziquantel treatment may be associated with a reduced risk to re-infection in a Ghanaian S. haematobium-endemic community
Source: PLoS Negl Trop Dis. 2022 Mar 9;16(3):e0010115. doi: 10.1371/journal.pntd.0010115 (PMC8906586; doi:10.1371/journal.pntd.0010115)
Supplement: S1 Text — Table A: List of threshold/cut-off values for IgG1, IgG4, and IgE responses to the S. haematobium antigens studied. Table B: Number of baseline responders to the S. haematobium antigens as compared to number of participants positive of S. haematobium infection at baseline. Table C: Information details on the 4 individuals who remained infected at Follow-Up (7 weeks post-PZQ treatment). Table D: Frequency of contact with, and extent of use of water body in the Abodom community. Fig A: Associations of Participant gender with S. haematobium (A) infection intensity and (B) Prevalence. The Wilcoxon paired matched sum sign rank test (A), and the Related Samples McNemar Test (B) were used to assess for significant differences in infection levels/prevalences over time for each group. The Mann Whitney U (A) and the χ2 tests were utilised to assess for significcant differences between groups at a particular time point. * is indicative of p-values < 0.05; *** is indicative of p-values < 0.001. Fig B: Associations of Participant gender with ShTAL1-IgE (A) percent response frequency and (B) levels at baseline and 7 weeks post-PZQ treatment. The Related Samples McNemar Test (A), and the Wilcoxon paired matched sum sign rank test (B) were used to assess for significant differences in infection levels/prevalences over time for each group. The X2 (A) and the Mann Whitney U tests were utilised to assess for significant differences between groups at a particular time point. ** is indicative of p-values < 0.01; and *** is indicative of p-values < 0.001 Fig C: Antibody levels to S. haematobium-specific antigens at baseline and 7 weeks post-Praziquantel (PZQ) treatment. (A) Box-and-whisker plots indicate differences in IgG1 and IgG4 titres at pre- and post-treatment to ShTAL1, ShAWA, and ShSEA. (B) Differences in IgE titres at baseline and 7 weeks post-treatment to S. haematobium-specific TAL1, AWA, and SEA. Lines within the boxes indicate median responses, whiles upper and lower whisker [file pntd.0010115.s001.docx]

**Manuscript Title:**

**Increased ShTAL1 IgE Responses Post-Praziquantel Treatment may be Associated with a Reduced Risk to Re-infection in a Ghanaian Schistosomiasis-endemic Community**

**SUPPORTING INFORMATION**

**Table A: List of threshold/cut-off values for IgG1, IgG4, and IgE responses to the *S. haematobium* antigens studied**

|  | **ShAWA IgG1 in ug/ml** | **ShAWA IgG4 in ug/ml** | **ShAWA IgE in ng/ml** | **ShSEA IgG1 in ug/ml** | **ShSEA IgG4 in ug/ml** | **ShSEA IgE in ng/ml** | **ShTAL1 G1 in ug/ml** | **ShTAL1 IgG4 in ug/ml** | **ShTAL1 IgE in ng/ml** |
| --- | --- | --- | --- | --- | --- | --- | --- | --- | --- |
| Mean | 0.18 | 0.01 | 0.18 | 0.10 | 0.07 | 0.38 | 13.99 | 0.06 | 0.71 |
| Standard Deviation | 0.55 | 0.00 | 0.29 | 0.00 | 0.11 | 0.75 | 27.41 | 0.07 | 2.39 |
| 3xSD | 1.6467 | 0.00 | 0.89 | 0.00 | 0.35 | 2.25 | 82.25 | 0.21 | 7.19 |
| Mean + 3xSD | 1.8310 | 0.01 | 1.06 | 0.10 | 0.42 | 2.62 | 96.25 | 0.28 | 7.89 |

**Table B: Number of baseline responders to the *S. haematobium* antigens studied as compared to number of participants positive for *S. haematobium* infection at baseline**

| Baseline  Parameter | **ShAWA_IgG1** | **ShAWA_IgG4** | **ShAWA_IgE** | **ShSEA_IgG1** | **ShSEA_IgG4** | **ShSEA_IgE** | **ShTAL1_IgG1** | **ShTAL1_IgG4** | **ShTAL1_IgE** |
| --- | --- | --- | --- | --- | --- | --- | --- | --- | --- |
| No. of Responders | 77 | 49 | 68 | 54 | 74 | 46 | 7 | 10 | 11 |
| No. of *S. haematobium* positives | 25 | 21 | 22 | 19 | 26 | 11 | 2 | 7 | 4 |

**Table C: Information details on the 4 individuals who remained infected at Follow-Up (7 weeks post-PZQ treatment)**

| **Participant ID** | **Age**  **(in years)** | **S. h. Infection intensity**  **(eggs/10ml urine)** | | **ShTAL1 IgE levels (ng/ml)** | | **ShTAL1 IgG4 levels (ug/ml)** | |
| --- | --- | --- | --- | --- | --- | --- | --- |
|  |  | **Baseline** | **Follow-Up** | **Baseline** | **Follow-up** | **Baseline** | **Follow-up** |
| 38 | 9 | 225 | 2 | 0.24 | 0.24 | 0.05 | 0.05 |
| 127 | 14 | 366 | 1 | 0.24 | 0.24 | 0.05 | 0.05 |
| 2 | 14 | 2 | 1 | 268.50 | 116.38 | 2,229.11 | 1,768.55 |
| 48 | 30 | 1 | 2 | 21.32 | 94.76 | 23.09 | 1,794.73 |
|  |  | **Geometric mean(GM)** | | **Median (min-max)** | | | |
|  |  | 20.14 | 1.41 | 10.78  (0.24-268.50) | 47.50  (0.24-116.38) | 11.57  (0.05 – 2229.11) | 884.30  (0.05 – 1794.73) |

**Legend:** Sh – *S. haematobium*; ShTAL1 IgE – *S. haematobium* Tegument allergen-like 1; IgE – Immunoglobulin E.

**Table D: frequency of contact with, and extent of use of water body in the Abodom community**

| Parameter | *S. haematobium* infection status | | Total  [N (%)] |
| --- | --- | --- | --- |
|  | Infected (n) | No eggs detected (n) | (N = 114) |
| Total number of participants | 27 | 87 | 114**^†^** |
|  |  |  |  |
| Main water source |  |  |  |
| stream/lake/pond | 27 | 68 | 95 (83.33) |
| well | 0 | 11 | 11 (9.65) |
| tap | 0 | 3 | 3 (2.63) |
| other | 0 | 1 | 1 (0.88) |
|  |  |  |  |
| Frequency of visit to stream/lake/pond |  |  |  |
| Never | 1 | 1 | 2 (1.75) |
| Once a month | 2 | 5 | 7 (6.14) |
| Two/more times a month | 5 | 11 | 16 (14.04)) |
| Once a week | 3 | 5 | 8 (7.02)) |
| Two/more times a week | 6 | 19 | 25 (21.93)) |
| Once a day | 0 | 3 | 3 (2.63) |
| More than once a day | 9 | 31 | 40 (35.09) |

**†** The total value against which the percentages were calculated (N = 114)


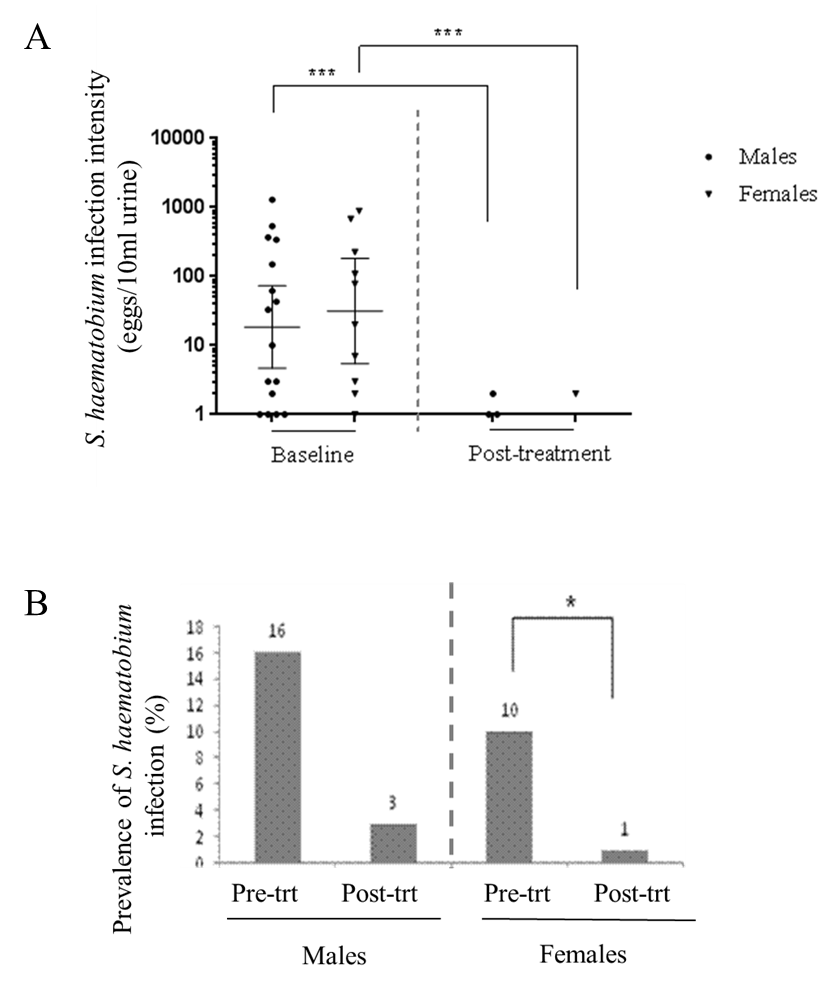
**Fig A:** Associations of Participant gender with *S. haematobium* (A) infection intensity and (B) Prevalence (N =26). The Wilcoxon paired matched sum sign rank test (A), and the Related Samples McNemar Test (B) were used to assess for significant differences in infection levels/prevalences over time for each group. The Mann Whitney U (A) and the ᵡ^2^ tests were utilised to assess for significcant differences between groups at a particular time point. * is indicative of p-values < 0.05; *** is indicative of p-values < 0.001.


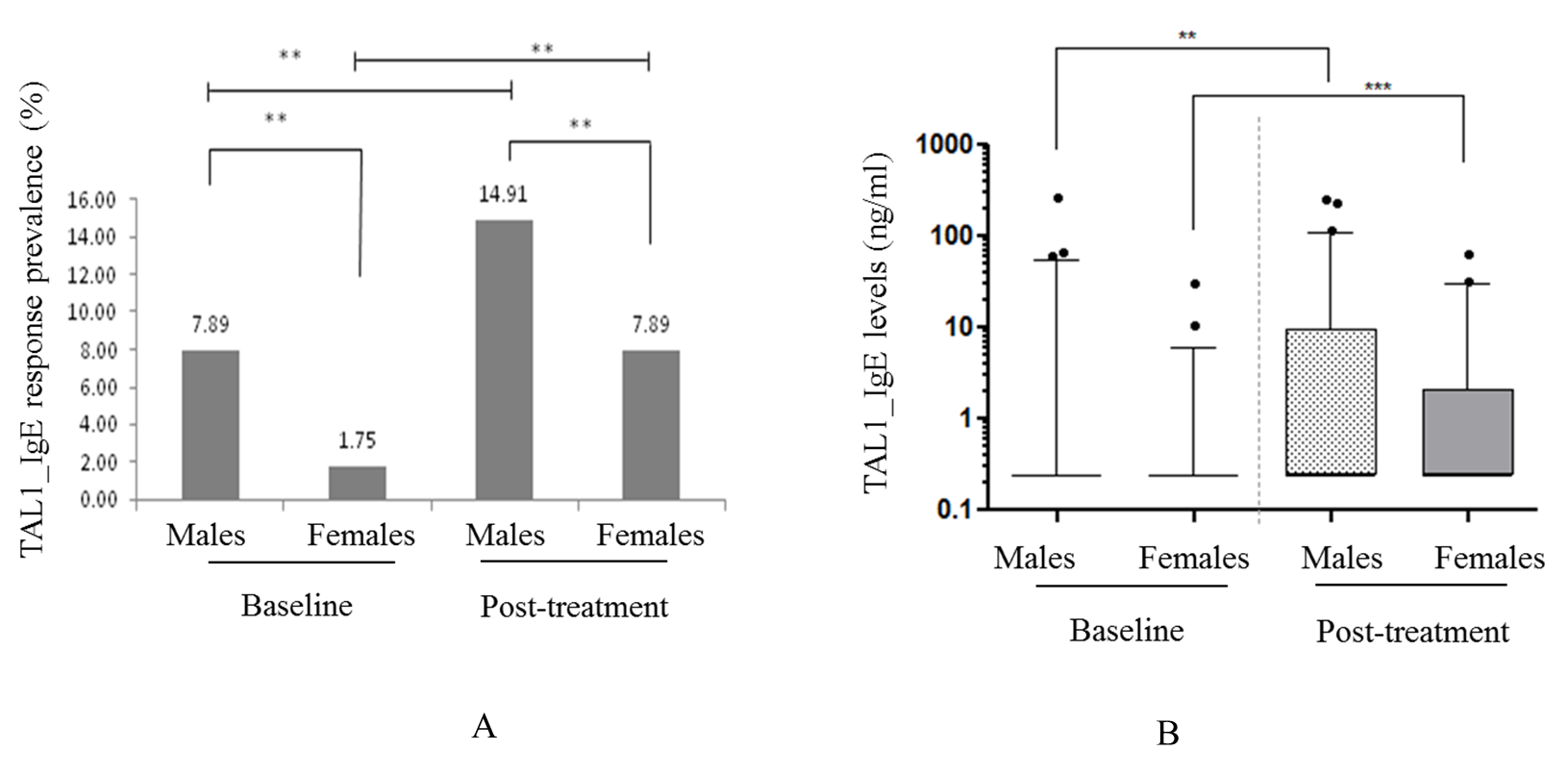


**Fig B:** Associations of Participant gender with ShTAL1-IgE (A) percent response frequency and (B) levels at baseline and 7 weeks post-PZQ treatment. The Related Samples McNemar Test (A), and the Wilcoxon paired matched sum sign rank test (B) were used to assess for significant differences in infection levels/prevalences over time for each group. The X^2^ (A) and the Mann Whitney U tests were utilised to assess for significcant differences between groups at a particular time point. ** is indicative of p-values < 0.01; and *** is indicative of p-values < 0.001


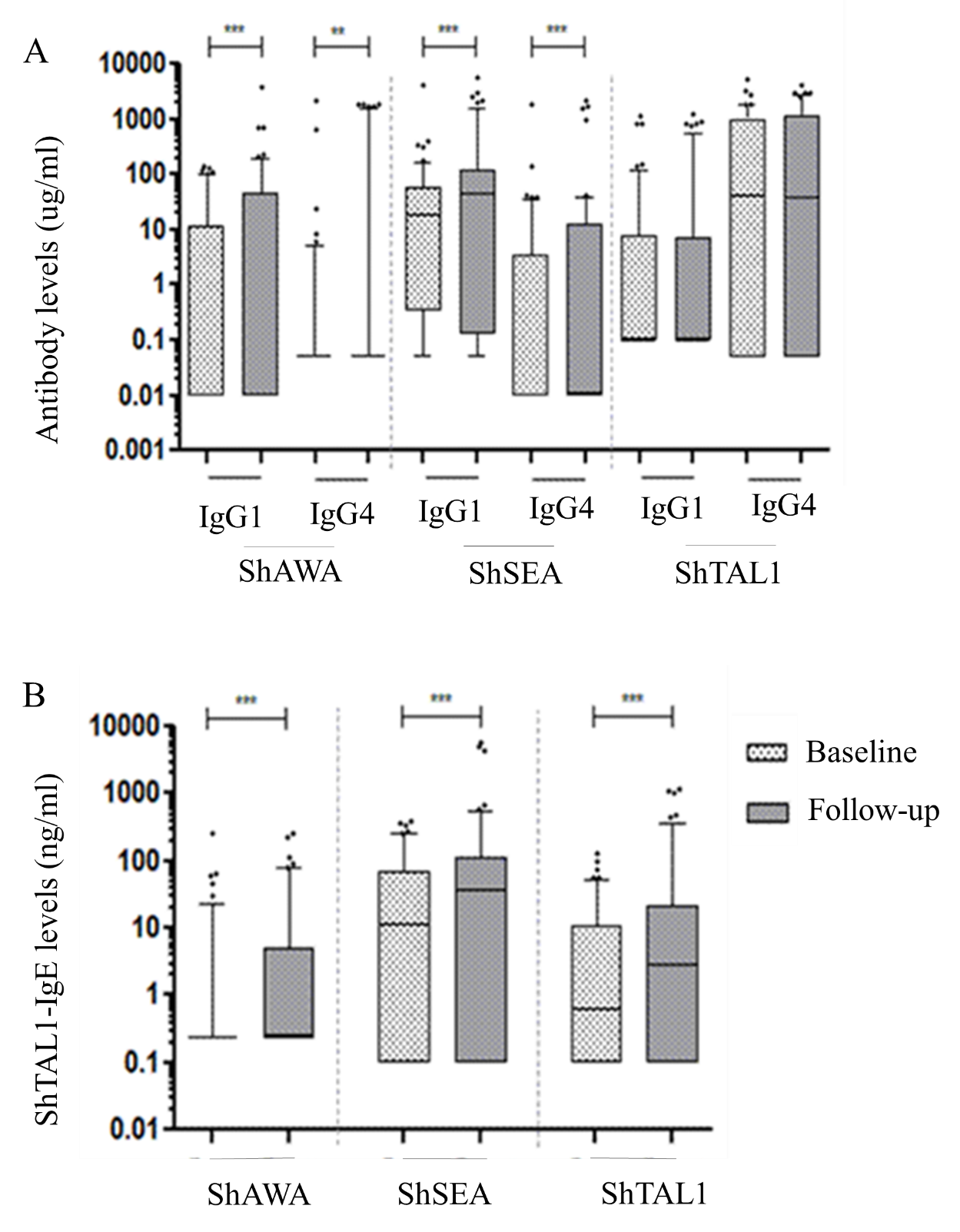


**Fig C:** **Antibody levels to *S. haematobium*-specific antigens at baseline and 7 weeks post-Praziquantel (PZQ) treatment (N = 114)**. (A) Box-and-whisker plots indicate differences in IgG1 and IgG4 titres at pre- and post-treatment to ShTAL1, ShAWA, and ShSEA. (B) Differences in IgE titres at baseline and 7 weeks post-treatment to *S. haematobium*-specific TAL1, AWA, and SEA. Lines within the boxes indicate median responses, whiles upper and lower whiskers are set at the 95^th^ and 5^th^ percentiles respectively. The Wilkoxon Matched-Pairs Signed-Ranks test was employed in determining p-values. ** is indicative of p-values < 0.01, whilst *** is indicative of p-values <0.001. ShTAL1 = *S. haematobium* (*Sh*)-specific tegumental allergen-like protein 1; ShAWA = *S. haematobium* (*Sh*)-specific adult worm antigen; ShSEA = *Sh*-specific schistosome egg antigen.


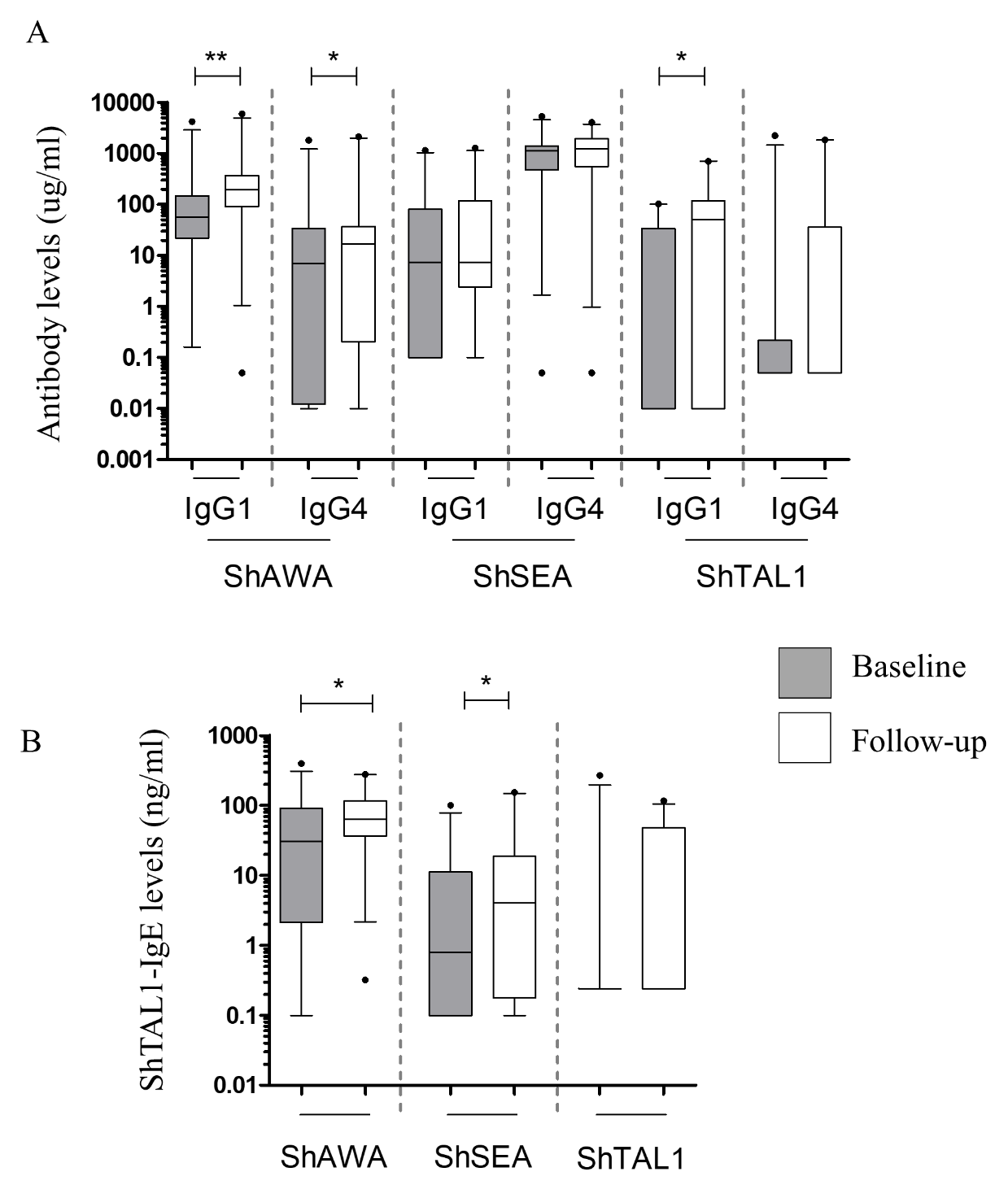


**Fig D:** **Antibody levels to *S. haematobium*-specific antigens at baseline and Follow-up (7 weeks post treatment with PZQ) for *S. haematobium*-infected participants who were egg-negative after PZQ treatment (N = 26):** (A) Box-and-whisker plots indicate differences in IgG1 and IgG4 titres at pre- and post-treatment to ShTAL1, ShAWA, and ShSEA. (B) Differences in IgE titres at baseline and 7 weeks post-treatment to *S. haematobium*-specific TAL1, AWA, and SEA. Lines within the boxes indicate median responses, whiles upper and lower whiskers are set at the 95^th^ and 5^th^ percentiles respectively. The Wilkoxon Matched-Pairs Signed-Ranks test was employed in determining p-values. * is indicative of p-values < 0.05, whilst ** is indicative of p-values <0.01. ShTAL1 = *S. haematobium* (*Sh*)-specific tegumental allergen-like protein 1; ShAWA = *S. haematobium* (*Sh*)-specific adult worm antigen; ShSEA = *Sh*-specific schistosome egg antigen.


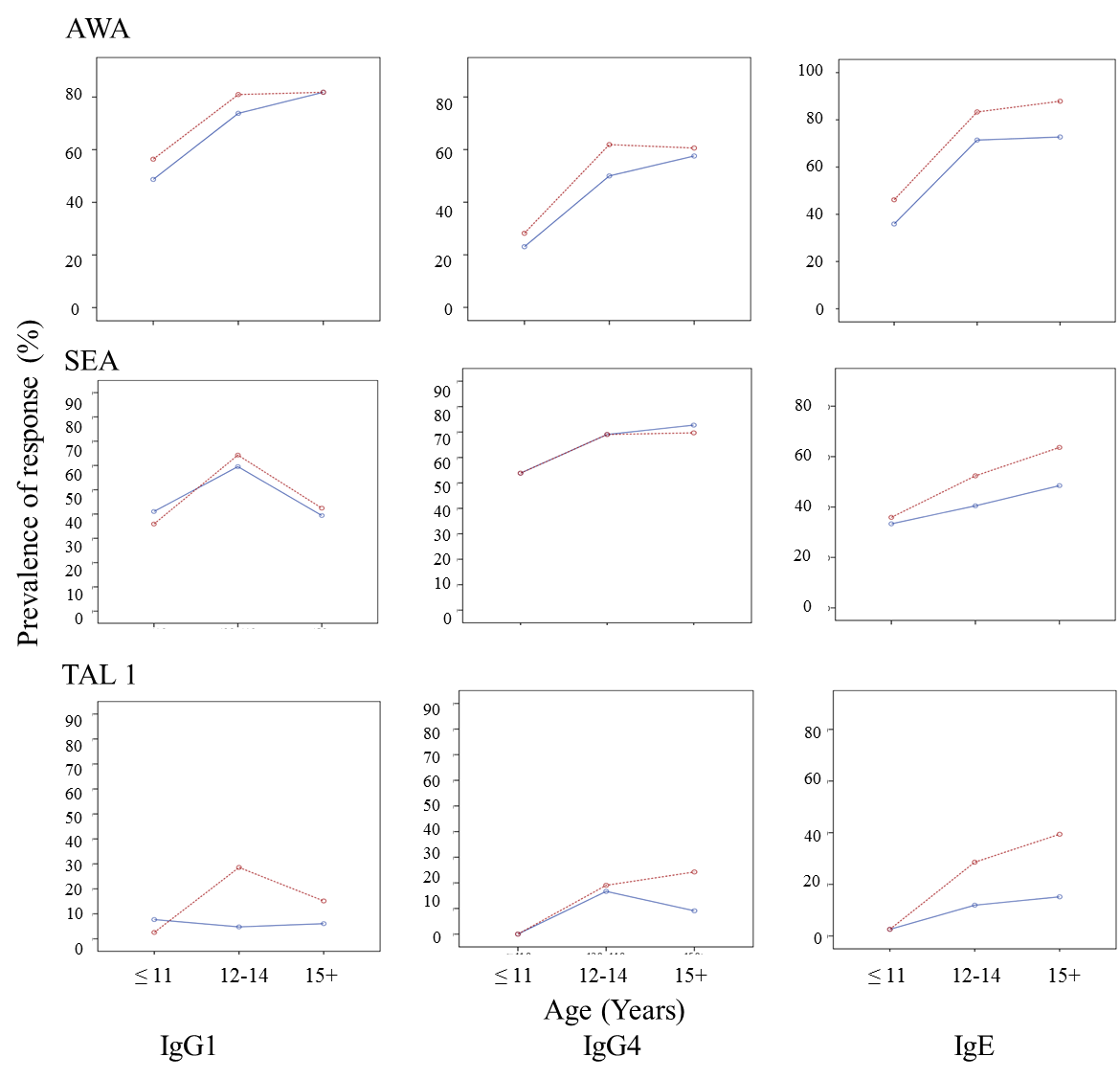


Legend:


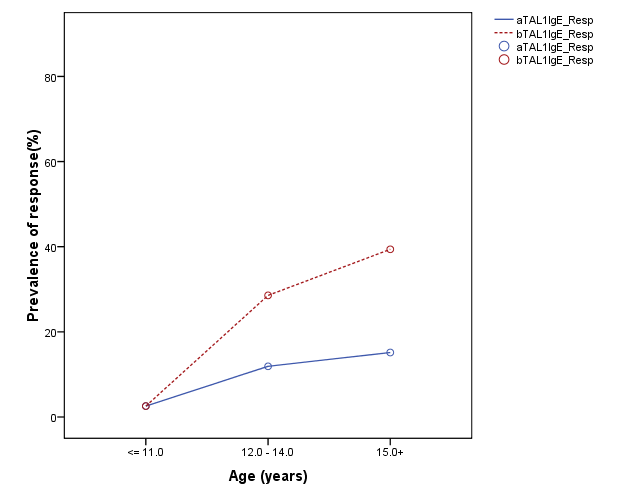

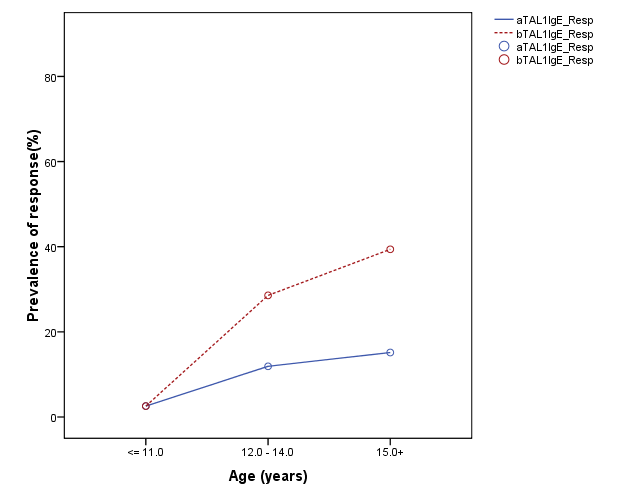


Baseline

Post-treatment

**Fig E:** Percent prevalences of IgG1, IgG4, and IgE antibody responses to *S. haematobium*-specific AWA, SEA, and TAL1 stratified by age at pre- and post- PZQ treatment. AWA = adult worm antigen; SEA = soluble egg antigen; TAL1 = tegumental allergen-like protein 1
